# Assessing the impact of blocking distal coronary sinus-left atrial muscular connection on inducible rate of atrial fibrillation and follow-up recurrence in persistent atrial fibrillation patients with different fibrotic degrees of left atrial: A retrospective study

**DOI:** 10.3389/fcvm.2022.987590

**Published:** 2022-10-13

**Authors:** FeiLong Zhang, JiChun Liu, Ping Fang, XiangHai Wang, JinFeng Wang, YouQuan Wei, Hao Yang

**Affiliations:** Department of Cardiology, The First Affiliated Hospital of Wannan Medical College, Wuhu, China

**Keywords:** persistent atrial fibrillation, fibrosis, low voltage area, distal coronary sinus musculature, induction of sustained atrial fibrillation

## Abstract

**Background:**

The musculature of the coronary sinus (CS), especially its distal connection with the post wall of the left atrial (LA), has been associated with the genesis and maintenance of atrial flutter (AFL) and atrial fibrillation (AF). However, the relative contributions of the distal coronary sinus (CSD)-LA connection to PersAF with various degrees of atrial fibrosis remain unknown.

**Objective:**

This study aimed to explore the different roles of blocking the CSD-LA connection in the induction of acute AF and middle-term follow-up of recurrence among PersAF patients with various degrees of LA fibrosis.

**Methods and results:**

A retrospective cohort of 71 patients with drug-refractory and symptomatic PersAF underwent ablation for the first time were studied. The population was divided into two groups according to disconnection of the CSD-LA or not. All patients enrolled accepted the unified ablation procedure (circumferential pulmonary vein isolation, non-pulmonary vein trigger ablation and ablation of the CSD-LA connection). Group A (*n* = 47) successfully blocked the CSD-LA electrical connection and Group B (*n* = 24) failed. Twenty-five patients could be induced into sustained AF in the Group A compared to 20 in the Group B (53.2 vs. 83.3%, *p* = 0.013). After a mean follow-up of 185 ± 8 days, 24 (33.8%) patients experienced atrial arrhythmia recurrences. The Group A had significantly fewer recurrences (25.5%) compared to Group B (50%). Meanwhile, in Group A, the ROC curve analysis suggested that in the case of blocking CSD-LA, low voltage area (LVA) of LA can act as a predictive factor for acute AF induction (AUC = 0.943, Cut-off = 0.190, *P* < 0.001) with sensitivity and specificity of 92.3 and 90.5%, and middle-term recurrence (AUC = 0.889, Cut-off = 0.196, *P* < 0.001) with sensitivity and specificity of 100 and 65.7%.

**Conclusion:**

Disconnection of CSD-LA could reduce the inducible rate of acute AF and the recurrences of atrial arrhythmia during middle-term follow-up. The PersAF patients with CSD-LA muscular connection blocked, experienced a higher acute AF inducible rate with larger proportion of LVA of LA (≥19%) and a higher recurrent rate of atrial arrhythmias with a larger proportion of LA fibrosis (≥19.6%).

## Introduction

The pulmonary veins (PVs) have been described to trigger atrial fibrillation (AF) since 1998 by Haïssaguerre et al. (1) and catheter ablation for AF has been considered a revolutionary strategy. For example, radiofrequency catheter ablation has been recommended as the optimal therapy to restore sinus rhythm (SR) in patients with drug-refractory and symptomatic AF. Regardless of paroxysmal AF (PAF) or persistent AF (PersAF), circumferential pulmonary vein isolation (CPVI) remains the cornerstone procedure. Meanwhile, the advocated additional ablation strategies of non-paroxysmal AF, such as substrate modification, linear ablation of the atrium, ablation of complex fractionated electrograms, non-pulmonary foci, and rotor ablation, have presented a revolutionary transition to acquire a higher long-term freedom rate of atrial tachycardias during follow-up (2, 3). Moreover, the DECAAF study (4) has illustrated that fibrotic disease of the atrium is associated with AF ablation outcomes. Thus, a personalized procedure according to the various degrees of atrial fibrosis should be considered. A previous study has also shown that individualized low voltage zone ablation based on high-density left atrial mapping can improve the outcomes of PersAF patients (5). This approach has also presented a similar result in the DECAAF study. Additionally, the Bipolar mapping is a convenient, efficient, and alternative way to quantify the fibrotic area of the LA (6). Besides, the CS musculature can be served as an anatomical substrate for AF and atrial flutter (AFL) (7). The presence of muscular connections between the CS and LA, coupled with rate-dependent unidirectional block, might act as a substrate for single or multiple reentries and atrial tachycardia (AT) induction (8). Importantly, during embryological development, the CS and the adjacent region of the LA bordering the mitral annulus conserve their muscular connections similar to the right horn of the sinus venosus and the primitive atrium (9). Although the epicardial bridge-coronary sinus muscular connections, particularly the distal one (CSD-LA) with left atrial (LA) post wall, are related to the initiation and perpetuation of AF/AFL and some cases have reported that the isolation of the CS from LA can further decrease the recurrence of AF during long term follow-up (10–12), a routine ablation procedure of the CSD-LA connection has not been yet integrated into additional ablation treatments. Hence, the epicardial structures contributing to AF might be underestimated. The progression from PAF to persistent/permanent AF is related to adverse events and mortality (2), and there are no definite and concrete additional strategies for different AF progression periods. Thus, imagining that the anatomical substrate, the CSD-LA connection, participates in AF progression, what should be done?

Herein, to provide more evidence of the associations of CSD-LA muscular connection with PersAF and its possible role in various degrees of the fibrosis of LA, we retrospected the different roles of blocking the CSD-LA connection in PersAF patients with different conditions of low voltage area (LVA) of LA. Overall, the inducible rate of sustained AF was recorded before and after the abaltion of the CSD-LA during SR and the middle-term results of follow-up were also analyzed.

## Materials and methods

### Population

This was a single-center, retrospective study that enrolled drug-refractory and symptomatic PersAF patients who accepted catheter ablation at the First Affiliated Hospital of WanNan Medical College from August 2020 to May 2022. The cohort was included in the study based on: (1) PersAF patients with no ablated history; (2) presence of CSD-LA electrical connection during sinus rhythm (SR) via electrophysiological methods; (3) CPVI followed by inducible AF after electrical cardioversion. Persistent AF was defined as AF continuously sustained for more than one week, including episodes terminated by cardioversion (drugs or electrical cardioversion) after ≥ 7 day (2). A total of 77 PersAF patients were enrolled, and six of them failed to complete the follow-up were excluded. According to the ablation results of CSD-LA connection, we divided these 71 patients into two groups. In Group A (*n* = 47) we successfully blocked the CSD-LA electrical connection and Group B (*n* = 24) failed. All 71 patients recruited for this study gave written informed consent to the unified ablation procedure (circumferential pulmonary vein isolation, non-pulmonary vein trigger ablation and ablation of the CSD-LA connection). The baseline clinical data of all patients are presented in Table 1. All patients were discontinued for antiarrhythmic drug therapy ≥5 half-lives before ablation.

**TABLE 1:** Clinical characteristics, procedural data of the study’s cohort (*n* = 71).

Variable	Overall (*n* = 71)	Disconnection of CSD-LA (*n* = 47)	Failed to block CSD-LA (*n* = 24)	*P*-value
**Clinical data**				
Age, year	64.3 ± 9.0	64.1 ± 8.0	64.1 ± 11	0.806
Female sex, *n* (%)	33 (46.5)	21 (44.7)	12 (50.0)	0.310
AF-during, months	41.8 ± 20.8	44.0 ± 23.4	37.4 ± 13.7	0.209
Smoking, *n* (%)	15 (21.1)	9 (19.1)	6 (25.0)	0.568
Alcoholism, *n* (%)	3 (4.2)	1 (2.1)	2 (8.3)	0.219
HF, *n* (%)	25 (35.2)	16 (34.0)	9 (37.5)	0.773
Obstructive sleep apnea, *n* (%)	16 (22.5)	11 (23.4)	5 (20.8)	0.806
eGFR, ml/min	90.4 ± 14.9	92.0.2 ± 16.1	87.2 ± 12.1	0.200
BMI, kg/m^2^	23.8 ± 2.6	24.2 ± 2.7	23.2 ± 2.2	0.128
Diabetes mellitus, *n* (%)	15 (21.1)	11 (23.4)	4 (16.7)	0.511
Hypertension, *n* (%)	34 (47.9)	23 (48.9)	11 (45.8)	0.804
**Echocardiographic data**				
LAD, mm	46.0 ± 5.1	46.2 ± 5.3	45.7 ± 4.6	0.695
LVEF, %	59.7.8 ± 6.2	60.0 ± 5.9	58.9 ± 6.8	0.497
**Procedural and endpoint data**				
Low voltage area of LA, %	19.8 ± 13.6	20.2 ± 12.9	19.1 ± 15.0	0.739
Follow-up time, days	185.1 ± 8.3	185.1 ± 7.8	185.0 ± 9.3	0.867

*BMI, body mass index; CSD-LA, distal coronary sinus-left atrium; eGFR, estimated glomerular filtration rate; HF, heart failure; LAD, left atrium diameter; LVEF, left ventricular ejection fraction. *Values are reported as medians and percentages. The LVA represents the proportion of the low voltage area of the LA.

### Electrophysiologic evaluations

Oral anticoagulation therapy was administered for at least 3 weeks before the ablation procedure and transesophageal echocardiography was performed within 3 day of the procedure to exclude atrial thrombus. All signals were stored on a recorder system (Labsystem Pro, Bard Electrophysiology, Lowell, MA, USA). Ablation and mapping accesses were established through bilateral femoral veins. Then, a decapolar catheter (2-8-2 mm interelectrode distance) was placed with its 9–10 electrodes at the CS ostium (CSO) through the left common femoral vein or the left subclavian vein. After the administration of intravenous heparin to reach an activated clotting time of 250–350 s, the LA was accessed using a 3.5 mm cold salt water-irrigated ablation catheter (Smarttouch SF) (Biosense Webster Inc.) and a multi-polar mapping catheter (PentaRay—20 electrodes with 2-6-2 mm spacing) via double trans-septal punctures through the right common femoral veins under fluoroscopy. Electroanatomic maps including anatomical maps, activation maps, and Bipolar maps were obtained with the 3-dimensional electroanatomic mapping system (CARTO 3; Biosense Webster, Diamond Bar, CA).

### Procedure

**FIGURE 1:**
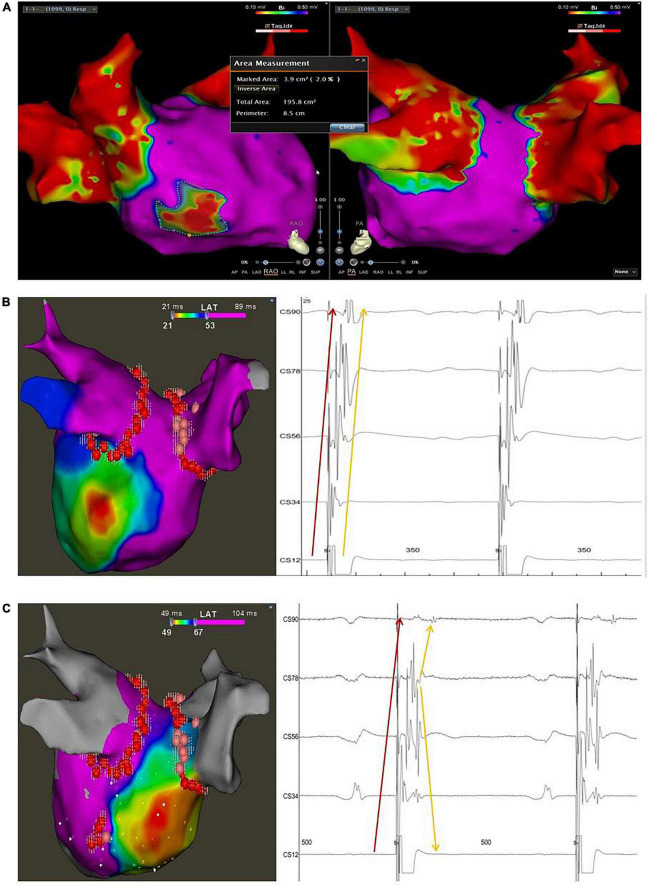
Typical case of the study with LVA < 10% and disconnection of the CSD-LA musculature. **(A)** The bipolar map of the LA after CPVI and before elimination of the muscular connection between the distal CS and LA was obtained with high-density mapping using a PentaRay catheter during SR; the marked area represents the low voltage area of the LA by the Area Measurement Module of the Carto 3 system; the total area represents the whole area of the LA including the PVS. **(B)**, left panel: activation map of the LA during distal CS pacing before elimination of the muscular connection between the distal CS and LA. **(B)**, right panel: intracardiac local electrograms recorded on the decapolar catheter during distal CS pacing before ablation at the connection between the distal CS and LA, presenting two discrete but parallel activated sequences from distant CS to proximity; the sharp electrograms pointed by the red arrow indicate the near field activation of the CS musculature, and the blunt electrograms pointed by the yellow arrow indicate the far-field activation of the LA. **(C)**, left panel: activation map of the LA during distal CS pacing after elimination of the muscular connection between the distal CS and LA; **(C)**, right panel: intracardiac local electrograms recorded on the decapolar catheter during distal CS pacing after blocking the connection between the distal CS and LA, presenting two discrete but intersectant activated sequences recorded on the polar CS; The sharp electrograms pointed by the red arrow indicate the near field activation of the CS musculature, which shows an activation sequence from distal to proximal; the blunt electrograms pointed by the yellow arrow indicate the far-field activation of the LA, which shows an activation sequence from proximal to distal. The pivot activation sequence recorded at the proximal polar CS (CS78) suggests the change of the earliest activation site of the LA from the posterior-lateral wall to the septum.

The procedure was conducted as follows: (1) After CPVI, the presence of CSD-LA electrical connections was identified by pacing maneuvers and the activation results of the LA post wall during SR. (2) Patients were divided into A and B groups according to ablation results of CSD-LA, and we enrolled the patients of inducible acute sustained AF. (3) The ablation of the CSD-LA was performed and the ablation results were verified. (4) The inducing methods were repeated and we acquired a inducing rate at the second time induction of AF. (5) Relevant parameters after ablation of the CSD-LA were evaluated using statistical methods. The acute recurrence of PersAF was measured by inducible sustained AF after the ablation procedure. Inducibility of sustained AF was defined as an AF persistent time ≥5 min via the same inducing maneuvers. The second inducing rate and the measurement of the relevant parameters didn’t represented the study’s endpoint. The endpoint was determined until the definite results of the follow-up. The method of validating the CSD-LA connection was presented in Figure 1.

### Circumferential pulmonary vein isolation

Further, an anatomical map of the LA was created using the mapping PentaRay catheter. The Ablation Index (AI)-guided CPVI was performed using radiofrequency applications with the CARTO3 system and Smarttouch SF. The radiofrequency energy for ablation was from 35 to 45 W with AI reaching the setting goals (400–500). The endpoint of this procedure was the bidirectional block between the pulmonary veins (PVS) and the LA. The AF was turned to SR by electrical cardioversion (EC), if not terminated after CPVI; and the documented non-PV triggers were also ablated. During or after the CPVI, Atrial flutter (AFL) or focal atrial tachycardia (AT) were ablated to SR when necessary.

### Presence of the distal coronary sinus-left atrial

**FIGURE 2:**
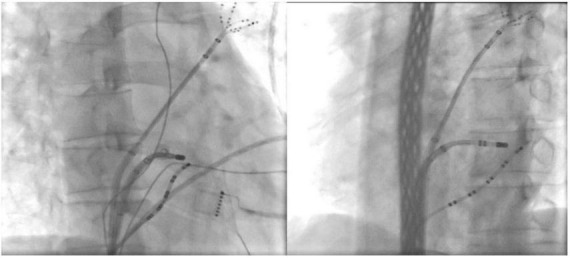
Ablation target of the CSD-LA electrical connection under fluoroscopic. *(Left) right anterior oblique view; *(Right) left anterior oblique view.

The presence of the CSD-LA muscular connection was validated by the following maneuvers: (1) Pacing the distal CS with minimum output, which could capture the distal musculature extended from the CSO but not the adjacent myocardium of the LA post wall. The local electrograms of the CS musculature and the adjacent atrial far-field potentials recorded on the CS deca-polar catheter were presented with the same activation sequence from the distal to the proximal CS (Figure 1B); or pacing the ablation catheter on the adjacent endocardium of the LA with minimum output, which could capture the myocardium of the LA but not the distal CS. The same activation pattern above appeared on the polar CS. The sharp electrograms of the distal CS were ahead of the atrial far-field potentials during distal CS pacing but reversed during ablation catheter pacing. (2) Activation map of the LA post wall with earlier sites located in the adjacent myocardium of the LA during distal CS pacing illustrated there as an electrical connection between the distal CS and LA post wall (Figure 2). The minimum pacing output varied for different patients, and it was identified by a decreasing output threshold until the distal CS could just be captured.

### Electroanatomic maps

The Bipolar map of the LA was acquired during SR with high-density mapping under the guidance of tissue proximity indication (TPI) using the PentaRay catheter and its threshold was set to 0.1–0.5 mv. The area of low voltage zones (<0.5 mv) was automatically calculated by using the Area Measurement Module of the CARTO 3 system. The total area of the LA was obtained after eliminating the unwanted area of the PVs. Then, the percentage of the low voltage area of the LA was presented (Figure 1A). Activation maps of the LA posterior wall during SR were performed twice, before and after ablation of the CSD-LA, by PentaRay at the minimum output pacing of the distal CS.

### Inducing methods

The induction of AF was performed twice, before and after the ablation of the CSD-LA musculature. The methods of AF induction included pacing stimulation and pre-induction medication. Infusion of high-dose isoproterenol and/or Adenosine Triphosphate were also applied for AF induction by intravenous drip or injection before or after pacing when necessary. Stimulation with incremental pacing (300, 250, 200, and 180 ms) or the shortest CL resulting in 1:1 atrial capture was also performed. The pacing was conducted on a deca-polar catheter from the proximal coronary sinus (CS90) and distal coronary sinus (CS12). Trains of 8–10 pacing stimuli were delivered during SR from each of the sites mentioned above. All inducing maneuvers were conducted unless sustained AF was induced.

### Ablation of the distal coronary sinus-left atrial

Ablation of the CSD-LA electrical connection was performed after activation mapping of the LA post wall during distal CS polar pacing. The ablation target was the earliest endocardial activated sites of the LA post wall, which was ablated with the nearly same ablation method 4–6 times. The radiofrequency application energy was delivered at 40 W with a contact force of 5–15 g and the AI for guiding ablation lesion was set for 400–450. The ablation target was not changed into the epicardial aspect if failed to endocardially block the CSD-LA (*n* = 21, contrast). After ablating the target, the validated maneuvers for checking the presence of the CSD-LA were repeated once to confirm the disconnection or not. Successful block of the distal CS to LA was confirmed by: (1) changed activation pattern and sequence on the polar CS (Figure 1C); (2) varied earliest activated sites of the LA from the distal CS to the LA roof or septum (Figure 1C).

### Statistical analyses

Continuous variables are presented as means ± standard deviations, and the categorical variables are expressed as counts (percentages). The χ2 test, independent samples Student’s *t*-test, and ROC curve analysis was used to evaluate differences in acute and prognostic parameter between two groups, and the cut-off proportion values of LVA for assessing the different roles of blocking CSD-LA electrical connection in acute AF induction and recurrence of atrial arrhythmias during follow-up. All analyses were performed using SPSS version 26 (SPSS Inc., IBM Corp., Chicago, IL).

## Results

### Demographic characteristics

A total of 71 PersAF patients were enrolled in the present study (46.5% of females; 64.3 ± 9.0 years; AF duration: 41.8 ± 20.8 months). Forty seven patients completed the disconnection of the CSD-LA (Group A) and 24 failed (Group B). The clinical characteristics of the two groups did not differ. All 71 patients completed the electrical isolation of pulmonary veins. Although all studied patients accepted the same methods of ablation, 24 failed to block the CSD-LA electrical connection. Hence, considering the complexity of CS distal musculature and the safety, the complete disconnection of the CSD-LA did not represent the endpoint of our study procedure.

### Inducibility of atrial fibrillation before ablation of the distal coronary sinus-left atrial

The PersAF was restored to SR by electrical cardioversion after pulmonary vein isolation. Sustained AF was inducible in all 71 patients before ablation of the CSD-LA, and these who could not be inducible were excluded.

### Inducibility of atrial fibrillation after ablation of the distal coronary sinus-left atrial

Sustained AF was still inducible in 45 (63.4%) patients after targeting the CSD-LA. In the Group A, 25 (53.2%) patients were induced into sustained AF after ablating the target, different from the Group B (20, 83.3%; *p* = 0.013) (Table 2).

**TABLE 2:** Endpoint data of the study’s cohort (*n* = 71).

Variable	Disconnection of CSD-LA, (*n* = 47)	Failed to block CSD-LA, (*n* = 24)	*P*-value
Inducible rate of AF, *n* (%)	25 (53.2)	20 (83.3)	0.013
Middle-term follow-up of atrial arrhythmia recurrences, *n* (%)	12 (25.5)	12 (50)	0.039

### Middle-term follow up of recurrences

After a mean follow-up of nearly 6 moths (185.1 ± 8.3 days), Among the 47 patients of Group B that presented successful disconnection of the CSD-LA, there were 12 (25.5%) patients experiencing the recurrences of atrial arrhythmia, vs. that of 24 patients of Group B which failed to disconnect the musculature experiencing 12 (50%) atrial arrhythmia recurrences (*p* = 0.039) (Table 2).

### Contributions of blocking the distal coronary sinus-left atrial in different low voltage area to acute atrial fibrillation induction

**FIGURE 3:**
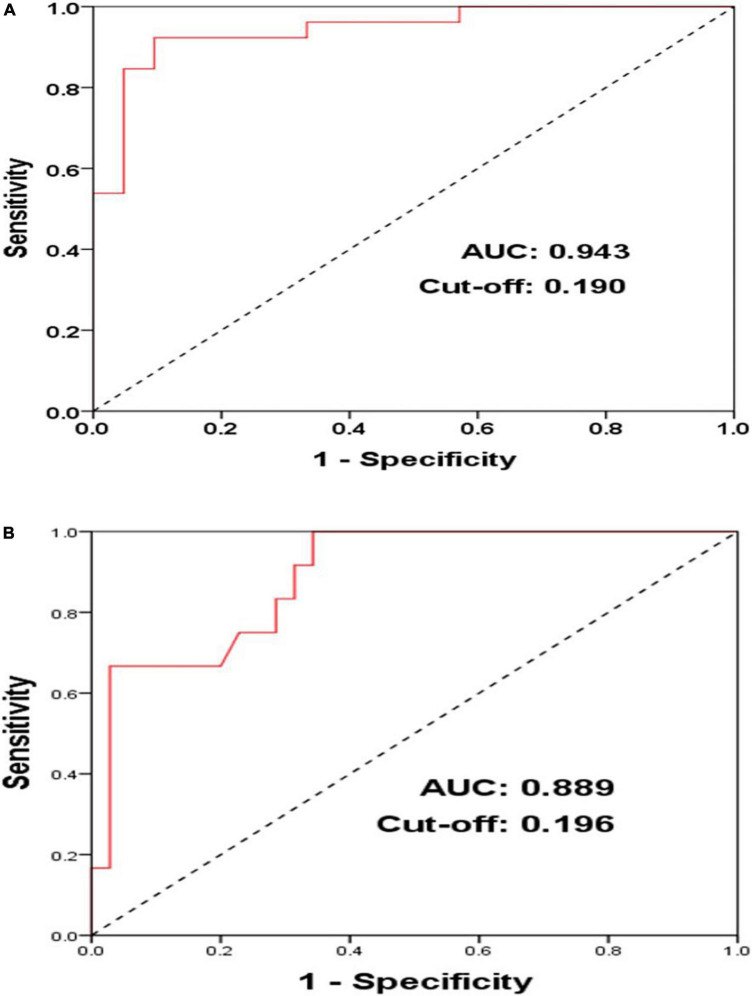
ROC (receiver operator curves) of acute AF induction **(A)** and middle-term follow-up of atrial arrhythmia recurrences **(B)** according to LVA in PersAF patients with blocked CSD-LA muscular connection (*n* = 47).

In Group A (*n* = 47), the ROC curve analysis suggested that in the case of blocking CSD-LA, LVA of LA can serve as a predictive factor for acute AF induction (AUC = 0.943, Cut-off = 0.190, *P* < 0.001) with sensitivity and specificity of 92.3 and 90.5% (Figure 3A).

### Contributions of blocking the distal coronary sinus-left atrial in different low voltage area to recurrences

Among the population with the CSD-LA electrical connection blocked (*n* = 47), the ROC curve analysis illustrated that in the case of blocking CSD-LA, LVA of LA can also serve as a predictive factor for middle-term recurrences of atrial arrhythmia (AUC = 0.889, Cut-off = 0.196, *P* < 0.001) with sensitivity and specificity of 100 and 65.7% (Figure 3B).

## Discussion

In the present study, we found a significant difference between the patients that successfully blocked the CSD-LA musculature (Group A) and those who did not (Group B) in the acute AF induction rate (53.2% vs. 83.3%, *p* = 0.013) and atrial arrhythmia recurrences of follow-up (25.5% vs. 50.0%, *p* = 0.039). These results might add further clinical evidences for the associations of the CS distal musculature with acute AF induction and its role in improving the outcomes of PersAF during follow up, which were coincident with previous studies conducted by Barletta et al. and Huang et al. (6, 8). Among the patients of Group A, the results of ROC curve analysis suggested that under the premise of blocking CSD-LA, LVA of LA below 19% was paralleled with lower acute AF induction (sensitivity and specificity of 92.3 and 90.5%) and LVA of LA below 19.6% accounted for a lower rate of atrial arrhythmia recurrences (sensitivity and specificity of 100 and 65.7%). This might indicate that blocking the CSD-LA connection would contribute more to decreasing acute AF induction and intensifying AF suppression and improving outcomes of catheter ablation in PersAF patients with better conditions of LA fibrosis. Furthermore, slow conduction and rate-dependent unidirectional block at the CS musculature and its bifurcations connected with the atria have been considered the origin of triggered activity (8, 13, 14). The CS and its musculature can develop unstable macro-re-entry and AF, which might be prevented by the isolation of CS muscular connections with the atrium. This has been demonstrated by results in which the CS can be a substrate of recurrent AF after PVI and that disconnection of the CS from the atrium might help prevent AF from recurrence and maintenance (15, 16).

The size of the LA, as a predictor of AF recurrence post-ablation, is associated with the degree of atrial structural remodeling and fibrosis (17), and the fibrosis of the atrium and complex fractional atrial electrograms have an important influence on the perpetuation and long-term outcomes of PersAF patients and CPVI might not be sufficient for AF patients with LVA of the LA > 10%. Besides, the CS is one of the targeting regions where complex fractionated atrial electrograms are frequently recorded (18, 19). For example, in a previous study with a canine AF model, atrial fibrosis was considered a vulnerable substrate for AF development (20). The electrical remodeling of AF is highly dependent on the slow conduction corridors and pivot points that can supply the functional substrate to form a localized re-entry for AF perpetuation (21). Additionally, AF itself can promote the fibrotic progression of the atrium to promote the maintenance and inducibility of AF (“AF begets AF”) (22, 23). Electrical and anatomic remodeling of the LA in AF patients can be manifested by low voltage zones, a large proportion of LVA, slow conduction regions, and an increased amount of complex electrograms of the atrium. Additionally, these abnormalities are pronounced in PersAF patients, suggesting that the remodeling degree of the atrium tends to be progressive (24). Although the significance of the AF inducibility after isolation of the PVs remains controversial, for patients with good condition in low voltage substrate, inducibility is a significant prognostic factor for long-term AF recurrence (25). Thus, acute inducible sustained AF is a limited but significant predictor for PersAF patients after the ablation procedure. Although enough evidence has shown that AF needs a trigger to be initiated and a vulnerable electrophysiological and/or anatomical substrate to be maintained, atrial fibrillation and fibrosis of the atrium is a complicated disease and its anatomic-functional basis is difficult to understand due to multiple aetiopathogenic mechanisms. Besides, the anatomic substrate contributes to the fibrillatory progression and maintenance of AF due to structural discontinuities and heterogeneous fiber orientation transmurally along the myocardial bundles (26).

To the best of our knowledge, this is the first study to investigate the different contributions of blocking the CSD-LA electrical connection to various degrees of LA fibrosis in first-time ablation PersAF patients. Overall, our results indicated that the actual role of the CS musculature in AF might be underestimated, and more attention should be paid to the CS anatomic substrate in patients with a lower proportion of low voltage zones of the atrium to consider the ablation strategy for PersAF.

### Clinical implications

Although CPVI has been considered the cornerstone catheter ablation therapy for drug-refractory and symptomatic AF, it has limited effects in non-paroxysmal AF. Advances regarding additional ablation strategies, such as linear ablation, substrate modification, complex fractionated atrial electrograms ablation, and rotor ablation, can reduce the recurrence of some PersAF patients, but their long-term outcomes remain not optimal (27–30). For PersAF, targeting the eccentric CSD–LA muscular connection in the early stage of atrial remodeling progression might comprehend a novel ablation strategy for reducing the acute recurrence of AF and promoting its outcomes of catheter ablation for PersAF. Furthermore, as an anatomical substrate associated with the initiation and maintenance of AF, blocking the connection between the CSD and LA might also prevent the atrium from fibrotic progression. Therefore, a more personalized ablation strategy for PersAF patients with early-stage fibrotic progression should consider the anatomic substrate.

## Limitations

This was a single-center study and had a limited sample size. However, the sample size was enough to demonstrate the differences in AF inducibility between the disconnection of the CSD-LA or not, and the different roles of the CSD-LA in various LVA of the LA. The concrete values of LVA with blocked CSD-LA needs further investigation and more cases and it will be studied in our future study. We just evaluated the patients that presented CSD-LA muscular connection and were inducible after CPVI during SR. Thus, the relationships between the patients without CSD-LA connection and AF induction remain unknown. Additionally, the anatomic complexity and diversity of the CS musculature and its distal extension, as well as the safety considerations, might have led to some of the failures during the ablation procedure of the CSD-LA (31).

## Conclusion

In conclusion, disconnection of CSD-LA could reduce the inducible rate of acute AF and the recurrences of atrial arrhythmia during middle-term follow-up. The PersAF patients with CSD-LA muscular connection blocked, experienced a higher acute AF inducible rate with larger proportion of LVA of LA (≥19%) and a higher recurrent rate of atrial arrhythmias with a larger proportion of LA fibrosis (≥19.6%). Thus, a more personalized ablation strategy for PersAF patients with early-stage fibrotic progression should take CSD-LA musculature into consideration.

## Data availability statement

The original contributions presented in this study are included in the article/supplementary material, further inquiries can be directed to the corresponding author/s.

## Ethics statement

The studies involving human participants were reviewed and approved by the First Affiliated Hospital of Wannan Medical College. The patients/participants provided their written informed consent to participate in this study.

## Author contributions

FZ drafted and corrected the manuscript. JL, YW, PF, JW, HY, and XW involved in investigation and data collection. All authors have read and approved the final manuscript for publication.

## Abbreviations

PersAF: Persistent atrial fibrillation
CS: coronary sinus
AFL: atrial flutter
AF: fibrillation
CSD: distal coronary sinus
LA: left atrial
LVA: low voltage area
CPVI: circumferential pulmonary vein isolation
SR: sinus rhythm
PVs: pulmonary veins
PAF: paroxysmal atrial fibrillation
AT: atrial tachycardia
EC: electrical cardioversion
AI: ablation index
TPI: tissue proximity indication.
